# Conformational Stability of the Hemagglutinin of H5N1 Influenza A Viruses Influences Susceptibility to Broadly Neutralizing Stem Antibodies

**DOI:** 10.1128/JVI.00247-18

**Published:** 2018-05-29

**Authors:** Wei Wang, Hyo Sook Song, Paul W. Keller, Esmeralda Alvarado-Facundo, Russell Vassell, Carol D. Weiss

**Affiliations:** a Laboratory of Immunoregulation, Division of Viral Products, Center for Biologics Evaluation and Research, U.S. Food and Drug Administration, Silver Spring, Maryland, USA; University of California, Irvine

**Keywords:** influenza hemagglutinin, stem antibodies, influenza neutralization, hemagglutinin stability, stalk antibodies, universal influenza vaccine

## Abstract

Vaccines that elicit broadly neutralizing antibodies to the conserved stem of hemagglutinin (HA) are being developed as universal influenza vaccines that protect against influenza across multiple years. However, different influenza virus strains, even those in the same subtype with identical stem sequences, can vary in susceptibility to broadly neutralizing stem antibodies, and the reasons are not understood. Here we studied potential mechanisms underlying the differing sensitivities of a panel of H5N1 HA pseudoviruses to broadly neutralizing stem antibodies. We found that greater HA conformational stability, as measured by thermal inactivation and pH triggering of conformational changes, correlates with reduced neutralization sensitivity and antibody binding to HA under neutral- and low-pH conditions. Our data indicate that the conformational stability of HA is an important attribute of susceptibility to broadly neutralizing stem antibodies and is influenced by residues outside the stem antibody epitopes.

**IMPORTANCE** The influenza virus surface glycoprotein hemagglutinin (HA) mediates virus attachment and membrane fusion between virus and host cells, allowing the viral core to enter the host cell cytoplasm for replication. Fusion occurs when HA undergoes low-pH-induced-conformational changes during endocytosis. Broadly neutralizing antibodies targeted to the conserved stem region of HA interfere with conformational changes required for fusion. Vaccines that elicit such antibodies are being developed as novel universal influenza vaccines for multiyear protection. We investigated why H5N1 HAs from different strains differ in their sensitivity to broadly neutralizing stem antibodies despite having conserved epitopes. We report that HA conformational stability due to residues outside the antibody binding site accounted for much of the variation in susceptibility to neutralization by stem antibodies. These findings highlight the importance of nonepitope residues in influencing neutralization sensitivity to stem antibodies and the complexities in developing universal vaccines targeting conserved epitopes in the HA stem.

## INTRODUCTION

Seasonal influenza virus infections cause severe respiratory illness in young children and adults, leading to the deaths of several hundred thousand people every year, with the majority occurring in the elderly (1–3). Humoral immune responses to the influenza hemagglutinin (HA) protein, the principal antigen in inactivated influenza vaccines, correlate with protection against influenza. Therefore, vaccination provides an important public health strategy.

HA is synthesized as a precursor polypeptide HA0 and is subsequently cleaved by cellular proteases to generate the HA1 surface subunit, forming the globular head domain that mediates binding to cell surface sialic acid receptors, and the HA2 transmembrane subunit, forming the major part of the stem region that mediates membrane fusion between viral and endosomal membranes during endocytosis (4–7). Most neutralizing antibodies (Abs) elicited by influenza virus infection or vaccination target the receptor binding site and surrounding residues on the head domain (8, 9). Viruses readily mutate these residues to escape antibody neutralization, leading to high sequence variability in the HA1 head domain. Thus, neutralizing antibodies targeting head epitopes are usually strain specific (10, 11). Due to the frequent emergence of influenza virus variants with mutations in HA that change antigenicity, influenza vaccines are reformulated annually to cover the dominant circulating strains.

Recently, broadly neutralizing antibodies targeting the HA stem were discovered (12–21). The HA stem region is highly conserved within influenza virus groups, as it is necessary for maintaining proper HA trimerization and mediating the fusion process through conformational changes. Thus, the stem region is an attractive target for developing universal influenza vaccines that elicit broadly neutralizing stem antibodies. However, different virus strains, even those within the same subtype and with identical stem epitopes, may have different sensitivities to stem antibody neutralization (18). We previously showed that HAs from different H5N1 strains differ in their susceptibilities to cross-neutralizing antibodies in human sera (22). The mechanisms behind these observed phenomena are not understood.

Conformational stability (flexibility) can be an important attribute of proteins involved in many biological systems. For example, the HIV envelope protein has been shown to reduce the accessibility of neutralizing antibodies via “conformational masking” (23). Similarly, the conformational flexibility of flaviviruses impacts viral susceptibility to antibody neutralization through changes in epitope accessibility (24–29). Monoclonal antibody binding to influenza HA also suggests that the HA trimers exist in multiple states (30). In this study, we investigated HA conformational flexibility and the relationship between the pH level of HA-mediated fusion and antibody binding as potential mechanisms underlying variations in the susceptibility of H5N1 HA to broadly neutralizing stem monoclonal antibodies (MAbs). Our data show that increased HA stability (decreased conformational flexibility) of HA is associated with viral resistance to broadly neutralizing stem antibodies. These finding have implications for vaccines targeting the stem of HA.

## RESULTS

### H5N1 HAs from different strains differ in their sensitivities to neutralizing stem antibodies.

Our earlier report showed that HAs from different strains of H5N1 viruses differ in their sensitivities to cross-neutralizing antibodies in human sera (22). To extend this observation, 16 pseudoviruses bearing HA from nine different H5N1 clades were tested for neutralization by well-characterized stem MAbs C179, CR6261, and FI6, which were all reported to neutralize H5N1 viruses. As shown in Table 1, HAs from some strains were more resistant to those stem MAbs whereas others, especially the HAs for A/Viet Nam/1203/2004 (VN/1203) and A/Indonesia/5/2005 (ID/5), were very sensitive.

**TABLE 1:** H5 virus sensitivity to stem antibodies

Clade	Virus strain	Stem antibody neutralization titer (μg/ml)		
		C179	CR6261	FI6-V3
0	A/Hong Kong/156/1997 (HK/156)	3.74	2.59	2.98
1	A/Viet Nam/1203/2004 (VN/1203)	0.09	0.06	0.11
1.1	A/Cambodia/R0405050/2007 (CB/R0405050)	>20	14.02	11.39
2.1.3.2	A/Indonesia/5/2005 (ID/5)	0.51	0.11	0.21
2.2.1	A/turkey/Turkey/1/2005 (TK/1)	>20	6.65	12.94
2.3.2.1	A/gray heron/Hong Kong/3088/2007 (HK/3088)	>20	>20	>20
2.3.4	A/Anhui/1/2005 (AH/1)	0.82	0.31	0.36
2.4	A/duck/Guangxi/13/2004 (GX/13)	>20	5.02	6.16
2.5	A/crow/Osaka/102/2004 (OS/102)	>20	5.66	6.22
3	A/duck/Hong Kong/2986.1/2000 (HK/2986.1)	>20	3.88	6.11
4	A/goose/Guiyang/337/2006 (GY/337)	>20	>20	>20
5	A/duck/Guangxi/1378/2004 (GX/1378)	10.69	2.66	3.46
6	A/duck/Hubei/wg/2002 (HB/wg)	3.84	1.58	1.53
7.1	A/chicken/Vietnam/NCVD-016/2008 (VN/NCVD-016)	1.45	0.55	0.74
8	A/chicken/Hong Kong/86.3/2002 (HK/86.3)	8.44	1.1	1.58
9	A/chukar/Shantou/4690/2003 (ST/4690)	>20	>20	>20

**FIG 1:**
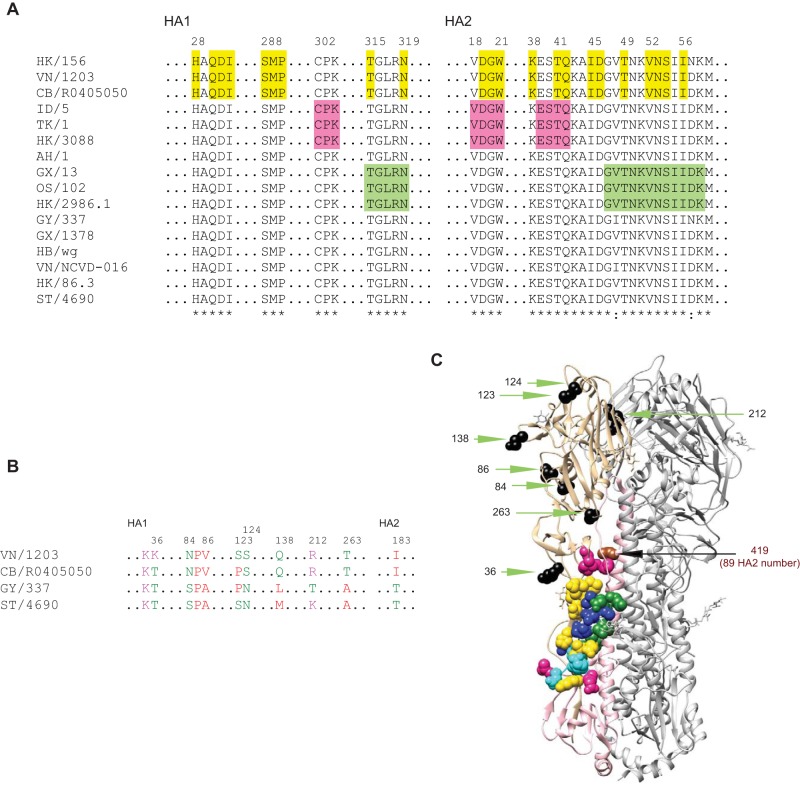
Stem antibody epitopes and sequences of different H5 HAs. (A) Stem antibody epitopes are conserved among different H5 HAs. (B) Residues in VN/1203 HA that differ from those in HA of at least two strains among CB/R0405050, GY/337, and ST/4690. (C) Stem antibody epitopes and different residues (shown in panel B) in VN/1203 HA trimer (PDB entry 2FK0). In panels A and C, the epitopes are indicated as follows: green, C179 epitopes; yellow, CR6261 epitopes; pink, FI6 epitopes. In panel C, the epitopes and residues are indicated as follows: blue, C179 and CR6261 overlap epitopes; cyan, CR6261 and FI6 overlap epitopes; sienna, residue 419 (HA2 number 89); black, different residues on HA1 shown in panel B. HK/156, A/Hong Kong/156/1997; VN/1203, A/Viet Nam/1203/2004; CB/R0405050, A/Cambodia/R0405050/2007; ID/5, A/Indonesia/5/2005; TK/1, A/turkey/Turkey/1/2005; HK/3088, A/gray heron/Hong Kong/3088/2007; AH/1, A/Anhui/1/2005; GX/13, A/duck/Guangxi/13/2004; OS/102, A/crow/Osaka/102/2004; HK/2986.1, A/duck/Hong Kong/2986.1/2000; GY/337, A/goose/Guiyang/337/2006; GX/1378, A/duck/Guangxi/1378/2004; HB/wg, A/duck/Hubei/wg/2002; VN/NCVD-016, A/chicken/Vietnam/NCVD-016/2008; HK/86.3, A/chicken/Hong Kong/86.3/2002; ST/4690, A/chukar/Shantou/4690/2003.

To investigate the factors underlying the different susceptibilities of various HAs to stem MAb neutralization, we examined stem sequences. On the basis of the published epitopes for MAbs C179, CR6261, and FI6 (12, 14, 18, 31–34), the sequences for the stem MAb epitopes among these H5 HAs were all conserved, except for the HA2 sequence of the C179 epitope in A/goose/Guiyang/337/2006 (GY/337) containing I48 and N57, that in A/chicken/Vietnam/NCVD-016/2008 (VN/NCVD-016) containing I48, and that in A/Hong Kong/156/1997 (HK/156) containing N57 that does not create a glycosylation motif (N-X-T/S) (Fig. 1A and C). Because HK/156 and VN/NCVD-016 contain these mutations and have much higher sensitivity to stem antibody neutralization than GY/337, it is likely that factors other than changes in the epitopes play a significant role in influencing susceptibility to stem antibodies.

### Stem antibodies differ in their levels of binding to different H5 HAs.

Next, we asked whether the differences in sensitivity to stem antibody neutralization were due to differences in the ability to gain access to the epitope in native HA. We selected several HAs, each with high, medium, or low sensitivity to stem antibody neutralization, and assessed stem MAb binding to HA pseudoviruses in an enzyme-linked immunosorbent assay (ELISA) format (Table 2). The dynamic range achieved using endpoint titers was modest but correlated well with sensitivity to neutralization by the stem MAbs. In particular, the least neutralization-sensitive HAs from the A/chukar/Shantou/4690/2003 (ST/4690), A/goose/Guiyang/337/2006 (GY/337), and A/Cambodia/R0405050/2007 (CB/R0405050) influenza virus strains had the lowest endpoint titers, while the most sensitive HAs from A/Viet Nam/1203/2004 (VN/1203), A/Anhui/1/2005 (AH/1), and A/Indonesia/5/2005 (ID/5) strains had the highest endpoint titers.

**TABLE 2:** ELISA endpoint titers of CR6261

Virus	ELISA endpoint titer (μg/ml)^*a*^
A/Hong Kong/156/1997 (HK/156)	0.06250
A/Viet Nam/1203/2004 (VN/1203)	0.03125
A/Cambodia/R0405050/2007 (CB/R0405050)	0.25000
A/Indonesia/5/2005 (ID/5)	0.03125
A/turkey/Turkey/1/2005 (TK/1)	0.12500
A/gray heron/Hong Kong/3088/2007 (HK/3088)	0.25000
A/Anhui/1/2005 (AH/1)	0.03125
A/duck/Guangxi/13/2004 (GX/13)	0.06250
A/crow/Osaka/102/2004 (OS/102)	0.12500
A/duck/Hong Kong/2986.1/2000 (HK/2986.1)	0.06250
A/goose/Guiyang/337/2006 (GY/337)	0.25000
A/duck/Guangxi/1378/2004 (GX/1378)	0.06250
A/duck/Hubei/wg/2002 (HB/wg)	0.06250
A/chicken/Vietnam/NCVD-016/2008 (VN/NCVD-016)	0.03125
A/chicken/Hong Kong/86.3/2002 (HK/86.3)	0.06250
A/chukar/Shantou/4690/2003 (ST/4690)	0.25000
A/Viet Nam/1203/2004 (VN/1203) K36T	0.03125
A/Viet Nam/1203/2004 (VN/1203) T263A	0.06250
A/Viet Nam/1203/2004 (VN/1203) L419I	0.06250

a The endpoint titer was defined as the lowest antibody concentration that gave an absorbance value of greater than 0.05 at 450 nm.

**FIG 2:**
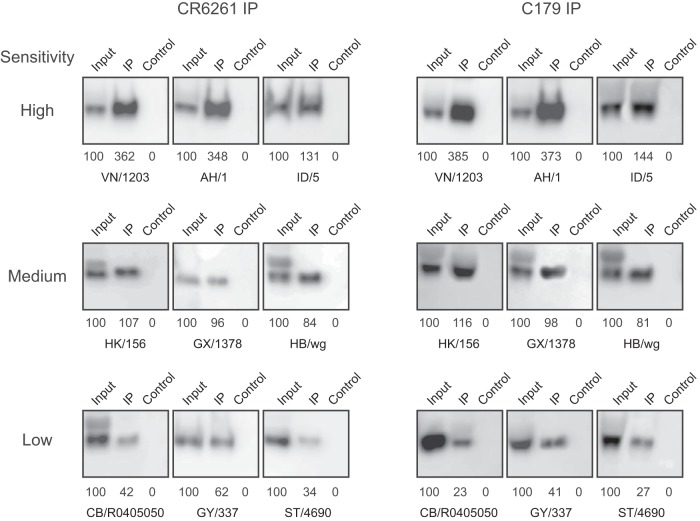
Stem antibodies vary in binding to different H5 HAs. H5 HAs with different sensitivities to stem antibody neutralization were immunoprecipitated by the use of CR6261 (left) and C179 (right) and analyzed by Western blotting with H5 HA1 antiserum. Percentages of the HA1 signal intensities relative to the total input of each HA are indicated below each blot. Input, 1/8 of total sample used for IP; IP, immunoprecipitation; Control, IP negative control with anti HIV gp41 antibody (Chessie 8).

To confirm the ELISA findings, we also performed immunoprecipitation assays, measuring the percentage of input HA pulled down by the stem MAbs. As shown in Fig. 2, CR6261 and C179 MAbs immunoprecipitated a large fraction of the input HA from the highly neutralization-sensitive HA pseudoviruses, while a much smaller fraction of the input HA was immunoprecipitated from the least neutralization-sensitive HA pseudoviruses. Together, the results from these binding studies suggest that accessibility of the epitope at neutral pH plays an important role in viral sensitivity to stem antibody neutralization.

### H5N1 HA conformational flexibility is associated with sensitivity to neutralizing stem antibodies.

**FIG 3:**
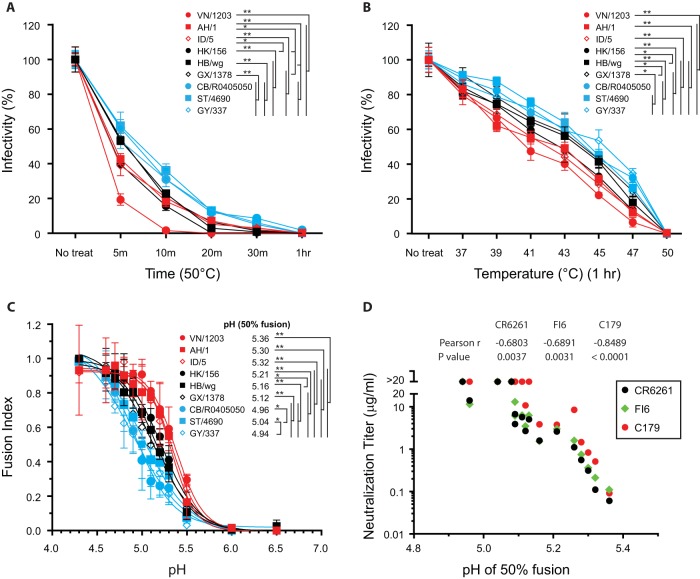
H5 HAs have different conformational flexibilities that are associated with different sensitivities to stem antibody neutralization. (A and B) The levels of infectivity of H5 HA pseudoviruses were compared after they were treated for different lengths of time at 50°C (A) and for 1 h at different temperatures (B). Viral infectivity after treatment was normalized to the infectivity of untreated virus. (C) Cell-cell fusion levels mediated by different H5 HAs at various pHs were compared by fusion index. For the fusion index comparisons, the fusion levels of each HA-induced fusion at various pHs were normalized to the maximum level of each HA-induced fusion. (D) Stem antibody neutralization titers for H5 HA pseudoviruses were correlated with the pH value that is needed for 50% fusion induction (compared to maximum fusion). Data are shown as means and standard deviations of results from three experiments. Line colors in panels A, B, and C are coded by color as follows: red, high sensitivity to stem antibody neutralization; black, medium sensitivity; blue, low sensitivity. *, *P* < 0.05; **, *P* < 0.01.

Since HA is metastable and needs to undergo pH-induced conformational changes to mediate virus entry, we surmised that differences in HA conformational flexibility influence the accessibility of stem epitopes for neutralizing antibodies. To test this hypothesis, we evaluated whether the HA pseudoviruses with different sensitivities to neutralization by stem MAb differed in their temperature stabilities. The levels of infectivity of HA pseudoviruses treated either at 50°C for 5 min to 1 h or at temperatures ranging from 37°C to 50°C for 1 h were compared with the levels determined for untreated HA pseudoviruses. All treatments reduced infectivity to different degrees among these HA pseudoviruses (Fig. 3A and B). Significantly, the HA pseudoviruses with high sensitivity to stem MAb neutralization had the least thermal stability, while the HAs with the lowest sensitivity to stem MAb neutralization showed the highest thermal stability. Since increases in temperature enhance the internal motion of proteins, these findings suggest that HAs with lower thermal stability have higher conformational flexibility that could affect exposure of new epitopes.

To further investigate the conformational flexibility (stability) of the various HAs, we measured the pH of fusion-inducing conformational changes in cell-cell fusion assays with different pH treatments. We reasoned that HAs with a higher fusion pH tolerance would have greater conformational flexibility for exposing residues for protonation. As shown in Fig. 3C, the HAs that were more resistant to stem MAb neutralization needed treatment at a lower pH to induce cell-cell fusion than the HAs with high sensitivity to stem MAb neutralization. The neutralization titers of stem MAbs against all tested H5 HA pseudoviruses correlated with the pH inducing 50% fusion (Fig. 3D). In summary, our results indicate that HA sensitivity to stem antibody neutralization is associated with HA conformational flexibility.

### Stem antibody binding to HA is stable under low-pH conditions.

**FIG 4:**
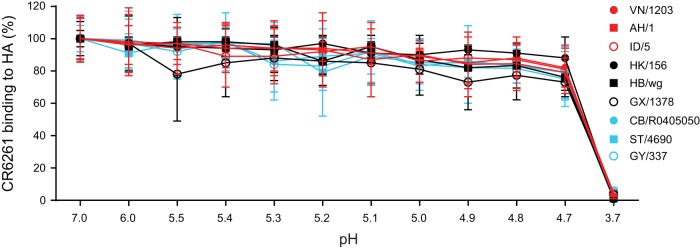
Stem antibody binding to HA is stable under a wide range of mildly acidic conditions (pH 4.7 to 7.0). CR6261 binding to HA coated on plates after treatment with pH 3.7 to 7.0 buffers was evaluated by ELISA. CR6261 binding to HA was normalized to the level of binding seen under conditions of pH 7.0 treatment. Data are shown as means and standard deviations of results from three experiments.

Because sensitivity to neutralization by the stem MAbs correlated with fusion pH, we questioned whether different rates of MAb dissociation from HA in endosomes could contribute to the neutralization susceptibility. If a MAb does not remain tightly bound to HA in endosomes to prevent fusion-inducing conformational changes, then HAs with lower pH thresholds of fusion might be more resistant to a MAb that dissociates before the HA mediates fusion. We tested this possibility in an ELISA format using different pH treatments. First, using rabbit antiserum cocktail against pan-H5 HAs, we confirmed that H5 HA pseudoviruses did not dissociate from coated ELISA plates after treatment with buffers with pH ranging from 4.6 to 7.0. We then performed stem MAb binding to HA pseudoviruses coated on plates at neutral pH before treating with buffers with pH values of 3.7 to 7.0 for 8 min followed by reneutralization to pH 7.0 and detection with secondary antibodies. Since coated HAs are not lost by treatment with buffers with pH ranging from 4.6 to 7.0, any changes of stem MAb binding under conditions of treatment at pH 4.6 to 7.0 would represent the effect of pH on stem antibody dissociation from HA. Figure 4 shows that there was little variation (*P* > 0.05) in the levels of MAb binding to the different HA pseudoviruses under a wide range of mildly acidic conditions (pH 4.7 to 7.0), suggesting that rates of stem MAb dissociation from HA under low-pH conditions of endosomes do not play a significant role in neutralization of susceptibility to stem MAbs.

### Nonepitope residues affect H5 HA flexibility and sensitivity to stem antibodies.

We next investigated which residues contributed to HA conformational flexibility and neutralization sensitivity to the stem MAbs by comparing sequences between A/Viet Nam/1203/2004 (VN/1203), which was the strain that was the most conformationally flexible and sensitive to stem antibodies in our assays, and A/Cambodia/R0405050/2007 (CB/R0405050), A/goose/Guiyang/337/2006 (GY/337), and A/chukar/Shantou/4690/2003 (ST/4690), which were the strains that were the most stable and resistant to stem antibodies. To increase the chances of identifying the residues that accounted for the phenotypic differences, we considered only those that were different from residues in VN/1203 and were present in at least two of the three resistant strains. All residues meeting these requirements were in the HA1 subunit, except residue 183 in the HA2 subunit, which is not present in the HA structure model (Fig. 1B and C). Among these HA1 residues, only residues 36 and 263 surround the epitopes targeted by stem antibodies (Fig. 1C). Therefore, we introduced K36T and T263A mutations into the VN/1203 HA.

To further investigate the relationship between HA stability and neutralization susceptibility to stem MAbs, we also introduced the L419I (418 in H3 HA numbering and 89 in HA2 numbering) mutation into the VN/1203 HA. Previously, we found that an L415I (418 in H3 numbering) change in seasonal H1N1 strains accounted for the resistance of H1 HA pseudoviruses to cross-neutralizing human sera (35). The same mutation was also reported to increase the stability of H5 HA (36), though 419I is not naturally present in the H5 sequences that we studied.

**FIG 5:**
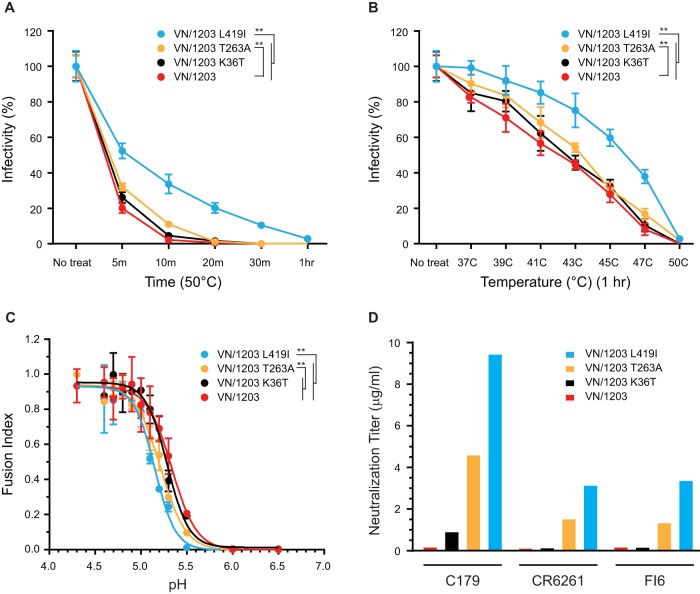
Nonepitope residues affect HA flexibility and sensitivity to stem antibody neutralization. (A and B) The infectivities of wild-type VN/1203 and the corresponding mutant HA pseudoviruses were compared after they were treated for different times at 50°C (A) and for 1 h at different temperatures (B). HA pseudovirus infectivity after treatment was normalized to the infectivity of the corresponding untreated HA pseudovirus. (C) Cell-cell fusion levels mediated by VN/1203 and its mutant HA at various pHs were compared by fusion index. (D) Stem antibody neutralization titers for VN/1203 and the corresponding mutant HA pseudoviruses. Data are shown as means and standard deviations of results from three experiments. **, *P* < 0.01.

As shown in Fig. 5A to C, compared to wild-type VN/1203, the T263A mutation conferred a modest but significant (*P* < 0.01) increase in both the thermal stability and pH stability of HA. The K36T mutation had little effect (*P* > 0.05) on stability, but the L419I mutation had a much larger effect (*P* < 0.01). In addition, the L419I and T263A mutations increased resistance to stem MAbs C179, CR6261, and FI6, with the greatest effect on MAb C179 (Fig. 5D). The K36T mutation increased resistance to MAb C179 only slightly and had no effect on the other MAbs. Similarly, T263A and L419I mutations modestly decreased binding to HA (Table 2). Overall, these results identified nonepitope residues affecting both conformational stability and sensitivity to stem MAb neutralization.

## DISCUSSION

Broadly neutralizing monoclonal antibodies to the conserved stem region of HAs are being used to guide the design of novel universal influenza vaccines. Despite conservation of stem epitopes, the sensitivities of strains to these antibodies vary widely. We investigated potential mechanisms underlying this variability in a large panel of HA from different H5N1 strains representing different H5 clades. Our studies showed that neutralization sensitivity to the stem antibodies correlates with HA conformational stability.

The liquid-like internal motions of proteins and the requirement of substantial plasticity in protein structure for many biological processes are well recognized (37–42). The influence of conformational fluctuations on epitope availability of envelope proteins of HIV, influenza virus, and flaviviruses has been reported previously (23, 29, 30). HA is metastable and needs to undergo pH-induced conformational changes to mediate virus entry. The resulting changes in the internal motions of HA can have consequences for exposure of stem epitopes. Once stem MAbs bind to HAs, the binding is stable across a wide range of low pH values that trigger fusion. Thus, it appears that the stem MAbs neutralize virus by first binding to HA at neutral pH and then remaining bound to HA during endocytosis to block the low-pH-induced HA conformation changes required for fusion.

Levels of HA stability differ among different strains. D94N (101 in H3 HA numbering), S217P (221 in H3 HA numbering), M226V (230 in H3 HA numbering), and L419I (418 in H3 HA numbering) have been reported to increase H5 HA stability (36). Among the H5 HAs that we investigated here, we noted that (i) the HAs of AH/1, TK/1, and OS/102 contain 94N, (ii) the HA of ID/5 contains 94S, (iii) the HA of GX/13 contains 94I, (iv) the HA of HK/156 contains 94N and 217P, and (v) the HA of HK/3088 contains 94N and 217I. All of those HAs are more stable than VN/1203 HA and are associated with less sensitivity to stem antibody neutralization. Consistent with previous reports, L419I (418 in H3 HA numbering) enhanced H5 HA stability (36) and reduced viral sensitivity to stem antibody neutralization (35).

Differences in HA stability are important for influenza virus host range, transmission phenotype, and pathogenic potential (43–46). A more stable HA (activation pH of ≤5.5) was previously shown to be necessary for pH1N1 influenza virus pathogenicity and airborne transmissibility in ferrets and to be associated with pandemic potential in humans (46). Similarly, increased H5 HA stability was reported to be associated with the enhanced H5 influenza virus transmission in ferrets (47–49). It will be important to assess the susceptibility of these airborne transmissible viruses to stem antibody neutralization. High HA stability is also critical for influenza vaccine development (50) because of the gradual degradation and aggregation of HA.

Our *in vitro* studies performed using the HA pseudoviruses and HA-mediated cell-cell fusion had several limitations. In particular, HA pseudoviruses based on lentiviral cores may differ from wild-type influenza viruses in the potential packing of and interactions between HA and neuraminidase (NA) on viral membranes. HA pseudoviruses are also restricted to single-cycle entry and neutralization. Importantly, we previously showed using reference sera that neutralization titers against H5 influenza HA pseudoviruses are similar to those measured in standard microneutralization assays (51). HA-mediated cell-cell fusion assays likewise do not precisely mimic influenza virus fusion in endosomes, and the use of a wide variety of methods to score fusion and report pH of fusion makes it difficult to compare pH measurements in the literature. For example, we report the pH of 50% maximal fusion using an enzymatic readout and a full range of pH values to generate a sigmoidal curve, while many others have reported the threshold pH for initiation of fusion and have used different readouts for fusion (36, 52–55). We also note that some studies report that NA can increase levels of HA-mediated membrane fusion and that syncytia were observed at higher pH when NA was expressed along with HA (54, 55). Further studies are needed to determine whether NA directly modulates HA stability.

In summary, our results show that residues outside the stem antibody epitopes can account for much of the variation of HA stability and susceptibility to stem antibody neutralization. Different combinations of residues in different HA contexts can affect HA stability and stem epitope exposure. These findings highlight the importance of nonepitope residues in influencing neutralization sensitivity to stem antibodies and the complexities of developing universal vaccines targeting conserved epitopes in the HA stem.

## MATERIALS AND METHODS

### Plasmids, cell lines, and antibodies.

β-Galactosidase (β-Gal) α subunit expression plasmid and 293T cells stably expressing β-Gal ω subunit (56) were provided by Nathaniel Landau (New York University, New York, NY). Broadly neutralizing stem MAbs CR6261, FI6, and C179, described previously (12, 14, 18), were obtained from the Vaccine Research Center (NIH, Bethesda, MD) and Yoshinobu Okuno (Kyoto, Japan). HIV-1 gp41 antibody (Chessie 8) (57) was obtained from the National Institutes of Health AIDS Research and Reference Reagent Program. As described previously (58), rabbit antisera against H5N1 HA1 were produced via immunization with A/Viet Nam/1203/2004 HA1 peptides (eENZYME). Rabbit antibodies against A/Viet Nam/1203/2004, A/Laos/3295/2006, and A/Indonesia/5/2005 HAs, generated by immunization with virus-like particles, were provided by Jerry Weir (FDA).

### HA pseudovirus thermal stability and neutralization.

H5 HAs from different clades as shown in Table 1 were used for pseudotyping lentiviral reporter vectors for producing HA pseudoviruses in 293T cells, as described previously (35, 51, 58). Briefly, 5 μg of pCMVΔR8.2, 5.5 μg of pHR′CMVLuc, 0.5 μg of HA, and 4 μg of A/California/04/2009 NA expression plasmids were included in the transfection. HA pseudotypes were collected 48 h posttransfection, filtered through a 0.45-mm-pore-size low-protein binding filter, and used immediately or stored at −80°C. For thermal stability studies, HA pseudoviruses were adjusted to have similar levels of infectivity at 37°C and were then treated for a specific time period at different temperatures. The levels of infectivity of untreated and treated HA pseudoviruses were compared. HA pseudovirus neutralization was tested as described previously (51). The antibody dilution causing a 95% reduction in the level of vector-expressed luciferase compared to control results was used as the neutralization endpoint titer (95% inhibitory concentration [IC_95_] neutralizing antibody titer) and calculated with nonlinear regression using GraphPad Prism. Data reported were from at least two independent experiments, with each sample run in duplicate.

### HA-mediated cell-cell fusion assay.

Cell-cell fusion was quantified using a reporter system based on β-galactoside (β-Gal) complementation (59). As previous described (60), 293T cells were transfected with wild-type or mutant H5 HA expression plasmids (pCMV/R-HA) and with β-Gal α subunit expression plasmids. The transfected 293T cells were detached using nonenzymatic cell dissociation solution (Sigma) 48 h posttransfection, and 6 × 10^4^ cells were then added to 6 × 10^4^ β-Gal ω subunit-expressing 293T target cells per well on a 96-well plate coated with poly-l-lysine solution. Cells were cocultivated for 3 h at 37°C and then treated for 5 min with phosphate-buffered saline (PBS)–0.1 M citric acid buffer at the desired pH. The treated cells were cultured in Dulbecco's modified Eagle medium (DMEM) overnight. Cell-cell fusion was then scored for β-Gal activity in coculture cell lysates using a Galacto-Star kit (Applied Biosystems, Carlsbad, CA), according to the manufacturer's instructions. Fusion levels were normalized as a fraction of the maximum fusion for each strain.

### Immunoprecipitation.

As previously reported (61), HA pseudoviruses were treated with 1% (final concentration) N-dodecyl β-d-maltoside (DDM) at 37°C for 1 h and incubated with stem MAb at 37°C for another 1 h followed by immunoprecipitation with protein G Dynabeads (Invitrogen) overnight at 4°C. After immunoprecipitation, the protein G beads were washed four times with 1% NP-40–PBS. The immunoprecipitation samples were resolved on SDS-PAGE and detected by Western blotting using rabbit H5 HA1 subunit antiserum.

### Hemagglutination and ELISAs.

The hemagglutination assay was performed in 96-well (V-bottom) plates by a standard method to determine numbers of HA units, as described in the WHO Manual on Animal Influenza Diagnosis and Surveillance (http://www.who.int/csr/resources/publications/influenza/en/whocdscsrncs20025rev.pdf), using 0.5% turkey red blood cells (Lampire Biological Laboratories, Pipersville, PA) suspended in PBS (pH 7.2). A standard ELISA was used to detect the binding of antibody to HA pseudovirus. Briefly, HA pseudoviruses containing 16 HA units were coated on ELISA plates. The virus-coated plates were blocked and then incubated with stem MAb or a rabbit antiserum cocktail against pan-H5 HA. The plates were then washed with PBS (pH 7.0) and incubated with peroxidase-conjugated secondary IgG (KPL, Gaithersburg, MD). Unbound secondary IgG was washed off as described above, signal was developed using TMB (3,3′,5,5′-tetramethylbenzidine) substrate, and the reaction was stopped with 1 N H_2_SO_4_ before recording the optical density at 450 nm (OD_450_).

### Computational analysis.

The HA structure comparison and the locations of the residues on HA were analyzed by the use of the UCSF Chimera program (https://www.cgl.ucsf.edu/chimera/) and Protein Data Bank (PDB) entry 2FK0 (62).

### Statistical analysis.

The correlation of neutralization titers with the pH of 50% fusion was evaluated with Pearson's *r*. The changes of thermal stability and pH for HA-mediated cell-cell fusion of each virus were compared by two-way analysis of variance (ANOVA), and the *P* values of the virus factor data are shown. *P* values of <0.05 in Pearson's *r* analysis and two-way ANOVA were considered statistically significant.
